# An open-access dataset of crop production by farm size from agricultural censuses and surveys

**DOI:** 10.1016/j.dib.2018.06.057

**Published:** 2018-06-23

**Authors:** Vincent Ricciardi, Navin Ramankutty, Zia Mehrabi, Larissa Jarvis, Brenton Chookolingo

**Affiliations:** a The Institute for Resources, Environment, and Sustainability, University of British Columbia, Canada; b School of Public Policy and Global Affairs, University of British Columbia, Canada

## Abstract

This dataset is a cross-country convenience sample of primary data measuring crop production and/or area by farm size for 55 countries that underlies the article entitled “How much of the world׳s food do smallholders produce?” (DOI: https://doi.org/10.1016/j.gfs.2018.05.002). The harmonized dataset is nationally representative with subnational resolution, sourced from agricultural censuses and household surveys. The dataset covers 154 crop species and 11 farm size classes, and is ontologically interoperable with other global agricultural datasets, such as the Food and Agricultural Organization׳s statistical database (FAOSTAT), and the World Census of Agriculture (WCA). The dataset includes estimates of the quantity of food, feed, processed agricultural commodities, seed, waste (post-harvest loss), or other uses; and potential human nutrition (i.e., kilocalories, fats, and proteins) generated by each farm size class. We explain the details of the dataset, the inclusion criteria used to assess each data source, the data harmonization procedures, and the spatial coverage. We detail assumptions underlying the construction of this dataset, including the use of aggregate field size as a proxy for farm size in some cases, and crop species omission biases resulting from converting local species names to harmonized names. We also provide bias estimates for commonly used methods for estimating food production by farm size: use of constant yields across farm size classes when crop production is not available, and relying on nationally representative household sample surveys that omitted non-family farms. Together this dataset represents the most complete empirically grounded estimate of how much food and nutrition smallholder farmers produce from crops.

**Specifications Table**

**Table:** 

Subject area	Agriculture, Food Security, Environmental Studies
More specific subject area	Crop Production, Crop Diversity, Farm Size, Smallholders
Type of data	CSV file
How data was acquired	All data were compiled via agricultural censuses or nationally representative household surveys.
Data format	Aggregated to sub/national level resolution.
Experimental factors	We describe the survey instruments used to build this harmonized dataset, and the methods of harmonization. We also test four aggregation assumptions we made with this dataset, including 1) using a constant yield across all farm size classes when crop production was not available, 2) using aggregate field size as a proxy for farm size, 3) relying on nationally representative household sample surveys that omitted non-family farms, and 4) crop species omission biases resulting from converting local species names to harmonized names. We also tested for regional biases resulting from our global convenience sample.
Experimental features	We describe key components of the data harmonization process and the dataset characteristics. Each of the four assumptions were tested in countries containing variables with both the assumption and the actual data. For example, we tested the constant yield bias in countries with datasets containing both the agricultural area and the actual crop production per farm size class. We then applied a constant yield across all farm size classes to the crop area variable and tested the difference between using the actual production versus the constant yield to calculate the production. Similar within country tests were conducted for each assumption.
Data source location	Sample containing 55 countries. See data coverage section for spatial coverage.
Data accessibility	Data accompanies article.

**Value of the data**

• The first open-access dataset containing food production by farm size at the global scale.
• Dataset can be used as a baseline for other global farm size datasets that do not contain direct measurements of smallholder food production.
• This dataset is harmonized across crop species, county, and year to link with the FAOSTAT and World Census of Agriculture databases.
• Contains 154 unique crop species, macro-nutrient conversion factors, and food, feed, and other production conversion factors that can be subset by farm size.
• This dataset is spatially explicit at the subnational level and is accompanied by a shape file with political boundaries for mapping.

## 1 Data

This dataset was built to provide estimates of the percentage of food produced by farms of different sizes globally. We constructed this dataset by harmonizing agricultural censuses and nationally representative household sample surveys that directly measured crop production and/or cropping area^1^ by farm size. This dataset is a convenience sample of 55 countries with 45 countries having sub-national resolution.

**Fig. 1:**
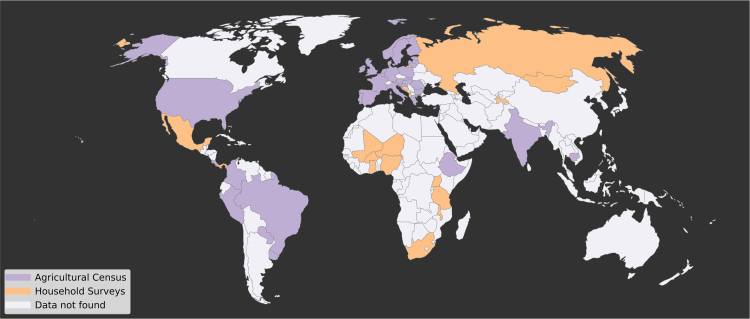
Map showing source of data derived from agricultural censuses (purple) or household surveys (orange) at the country level.

Our dataset captures ~51.1% of global crop production and ~52.9% of global cropland area (i.e., arable land and permanent crop area as reported in the Food and Agricultural Organization׳s statistical database (2017) [FAOSTAT hereafter]) [1]. The primary sources are agricultural census data (i.e., the majority of which used exhaustive sampling of the farming population, but not all response rates were 100%) or nationally representative sample surveys (i.e., with randomly stratified sampling of households in a country). These data were available at either the aggregated level by administrative unit (34 countries) or at the non-aggregated, microdata level where data are available as anonymized individual household level records (21 countries, of which 18 were sample surveys and 3 were complete agricultural censuses) (Fig. 1). We document the source information, detail the methods for building this dataset, and describe its characteristics in this article to enable its use by the research community.

This database was harmonized across countries, 154 crop species, and farm size categories. Crop species and country names were matched with FAOSTAT by year to integrate with its extensive variable lists. The median year of the source data was from 2013, with the oldest source dataset from 2001 and the newest from 2015; each administrative unit contains data for the most recently available time point. We harmonized the farm size categories to match the World Census of Agriculture (WCA) farm size categories: 0 to 1 ha, 1 to 2 ha, 2 to 5 ha, 5 to 10 ha, 10 to 20 ha, 20 to 50 ha, 50 to 100 ha, 100 to 200 ha, 200 to 500 ha, 500 to 1000 ha, and above 1000 ha.

We ran into several methodological issues when harmonizing the underlying data needed to construct this dataset. In this article, we outline the assumptions made, and test the bias of these assumptions, such as applying constant yields across farm size classes to estimate production when only cropping area was available (representing ~60% of our data), omitting non-family farms when relying on household sample surveys (22.5% of our data), using aggregated plot size as a proxy for farm size (~5% of our data), and omitting crop species that we were unable to be harmonize across countries or with the FAOSTAT crop species list.

In this article, we also provide details on the data collection and inclusion process, summary statistics, spatial coverage, and provide sensitivity tests and/or detailed explanations of each of the data harmonization assumptions we made. Our goal is to be transparent about our dataset׳s limitations, offer insight for other data harmonization projects relying on these same biases, and offer guidance for people wishing to use this data in their own work.

## 2 Experimental design, materials, and methods

### 2.1 Methods for data selection

#### 2.1.1 Inclusion criteria

We prescribed four inclusion criteria for this project. First, datasets needed to contain variables for farm size (where farm size was not available we relied on aggregate field size)cross-tabulated with production per crop or cropping area per crop. Second, datasets needed to be nationally representative. Agricultural censuses or household sample surveys were used only when their sampling methodology was transparent and/or these datasets were used by the country׳s government for official statistics. We required the household surveys’ sampling designs to be transparent, randomized at the appropriate administrative unit, and to provide sampling weights and expansion factors with details on their creation and intended application. Third, national numbers calculated from these datasets needed to be comparable with official national statistics. For many agricultural censuses, the sampling design and response rates were not available. Fourth, we only focused on surveys which included disaggregated data on crop species so that they could be matched to FAOSTAT crop names and item codes. No aggregate categories were used (e.g., ‘roots and tubers’ or ‘fruit and vegetables’).

We systematically searched several locations for agricultural datasets to compile our dataset. These sources included the World Bank microdata archives, EarthStat metadata, Living Standards Measurement Study (LSMS) surveys, and the Accelerated Data Program (see Table 1 for full data repository list). We conducted our search on a per country basis either through each data archive׳s search capabilities where available, detailed search of each data archive׳s metadata, or via web-scraping the archive to identify pertinent variables. Due to the multilingual nature of the datasets, variables were translated using the Google Translate Application Programming Interface (API) and we cross-checked any ambiguous or unknown colloquial crop name against several sources [2], [3] and/or with colleagues who work in each region of interest. For each country in each data archive, we searched for variables that directly linked ‘farm size’ or ‘plot area’ with ‘production’ or gross ‘plotted׳/׳cropped׳/׳planted׳/harvested׳ area by ‘crop type’. If there were multiple eligible datasets available per country, we included the most recent year. Nearly all the source data were freely obtained and all are used according to their user agreements.

**Table 1:** Data repositories.

Name	Region	Link
Accelerated Data Program	Global	http://adp.ihsn.org/country-activities
Africa Bank Group	Africa	https://www.afdb.org/en/knowledge/statistics
African Growth and Development Policy	Africa	http://www.agrodep.org/datasets
Consultative Group to Assist the Poor	Global	http://www.cgap.org/data
DataFirst	Africa	https://www.datafirst.uct.ac.za
Earthstat	Global	http://www.earthstat.org/
Harvard׳s Dataverse	Global	https://dataverse.harvard.edu/
Harvest Choice	Global	https://harvestchoice.org
International Food Policy and Research Institute	Global	http://library.ifpri.info/data
International Household Survey Network	Global	http://catalog.ihsn.org/index.php/catalog
Living Standards Measurement Study	Global	http://www.worldbank.org/en/about/unit/unit-dec
Prism	Oceania	http://pdl.spc.int/index.php/catalog
UNICEF Multiple Indicator Cluster Surveys	Global	http://mics.unicef.org/surveys
World Bank׳s microdata repo	Global	http://microdata.worldbank.org
World Food Program	Global	http://nada.vam.wfp.org/index.php/catalog
World Food Programme׳s Survey Data Portal	Global	http://nada.vam.wfp.org

Of the censuses that we included and had detailed sampling information (25 countries), 15 countries relied on either an exhaustive sampling design or a design that was exhaustive for farms with a set number of employees and/or annual revenue and a sample survey for smaller farms. Of the exhaustive censuses, there was a median response rate of 80%; the remaining censuses relied on stratified randomized sampling and applied resampling weights and expansion factors before making their aggregated data available (see dataset׳s metadata).

#### 2.1.2 Farm size harmonization

**Fig. 2:**
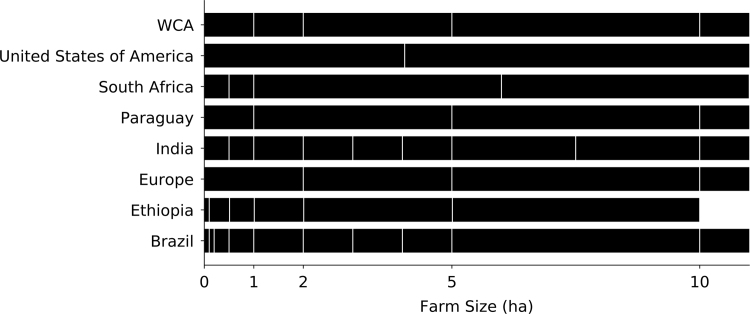
Farm size harmonization. Countries shown are where the given farm size classes were harmonized against the World Census of Agriculture (WCA) farm size classes. European countries from the Eurostat database had common farm size classes and are grouped together. Any country not shown contained directly matched farm size classes to the WCA. Since the majority of re-grouping occurred < 10 ha, the remaining farm size classes are not shown.

For tabulated census data, we made adjustments in order to match the census data to the farm size classes that were reported in the WCA in order to enable consistent analyses across all countries. In some instances, census data farm size classes could simply be aggregated to match those reported in the WCA. In other instances, census data classes needed to be disaggregated into two or more WCA classes. For countries that had both tabulated census data and microdata available, the available area data in the microdata was aggregated into WCA classes, and the proportion represented by each class was used to distribute census data. For countries that had agricultural area by farm size class reported that differed from the classes in the WCA, the proportion of area in each class was used to disaggregate subnational census data classes where necessary. For example, Paraguay reported a farm size class of 1–5 ha, whereas the WCA reported classes 1–2 ha and 2–5 ha. The total area in the 1–5 class was split between the two smaller classes based on their relative size, so 25% of area was assigned to the 1–2 ha class, and 75% of area was assigned to the 2–5 ha class. For all other countries, the simplest solution was to aggregate classes to match the WCA farm size classes. There were instances where two different methods were used for the same country. Additionally, there were situations were a country׳s largest farm size class differed from the WCA׳s largest farm size class, yet encompassed all farm sizes over a certain threshold. For example, in countries that only reported the largest farm size class to be over 100 ha, all farms over 100 ha would be entered into the WCA׳s corresponding 100-200 ha class. While this is a limitation of the data harmonization process, we were not able to assume a distribution for a country׳s largest farm size class through which we could dissagregate into several of the larger WCA classes. Fig. 2 shows a subsection of reported farm size classes for tabulated census data (all European countries reported in Eurostat had the same classes, represented by the Europe category in Fig. 2). The WCA classes, which were used in our analyses, are also shown. Corrections were made for the following countries: Austria, Belgium, Brazil, Bulgaria, Croatia, Cyprus, Czech Republic, Denmark, Estonia, Ethiopia, Finland, France, Germany, Greece, Hungary, Iceland, India, Ireland, Italy, Latvia, Lithuania, Luxembourg, Malta, Montenegro, Netherlands, Norway, Paraguay, Poland, Portugal, Romania, Slovakia, Slovenia, South Africa, Spain, Sweden, Switzerland, United Kingdom, United States of America (Fig. 2).

#### 2.1.3 Construction of conversion factors

Conversion factors for kilocalories, fats, and proteins (in grams per capita) and for the percentage of each crop grown for food, animal feed, processed commodity, seed, and wastage due to transportation and storage (but not home consumption) were calculated using FAOSTAT. FAOSTAT provides actual values for each of these variables at the national level per year with detailed definitions. For example, if a country produced soybeans in a given year, we took the ratio of the amount of soybean production allocated towards food divided by the total soybean production in that country to obtain the conversion factor for that country and year. We would repeat for feed, processed goods, seed, and waste, then apply these conversion factors to the amount of production each farm size produced per administrative unit in that country, and for each crop type. Hence, each estimate for these macro-nutrient and production variables assumes the national allocations are homogeneous across all administrative units and across all farm sizes. This is a largely untested assumption, and to our knowledge there are no sub-national datasets nor farm size specific datasets covering these variables, and therefore the bias introduced by it is unknown (unlike for some other assumptions for which we were able to estimate bias, see Section 4). To enable future researchers to accommodate adjusting these conversion factors, we provide the actual amount of production per farm size per administrative unit in addition to the conversion factors and converted values.

#### 2.1.4 Dataset descriptive statistics

**Fig. 3:**
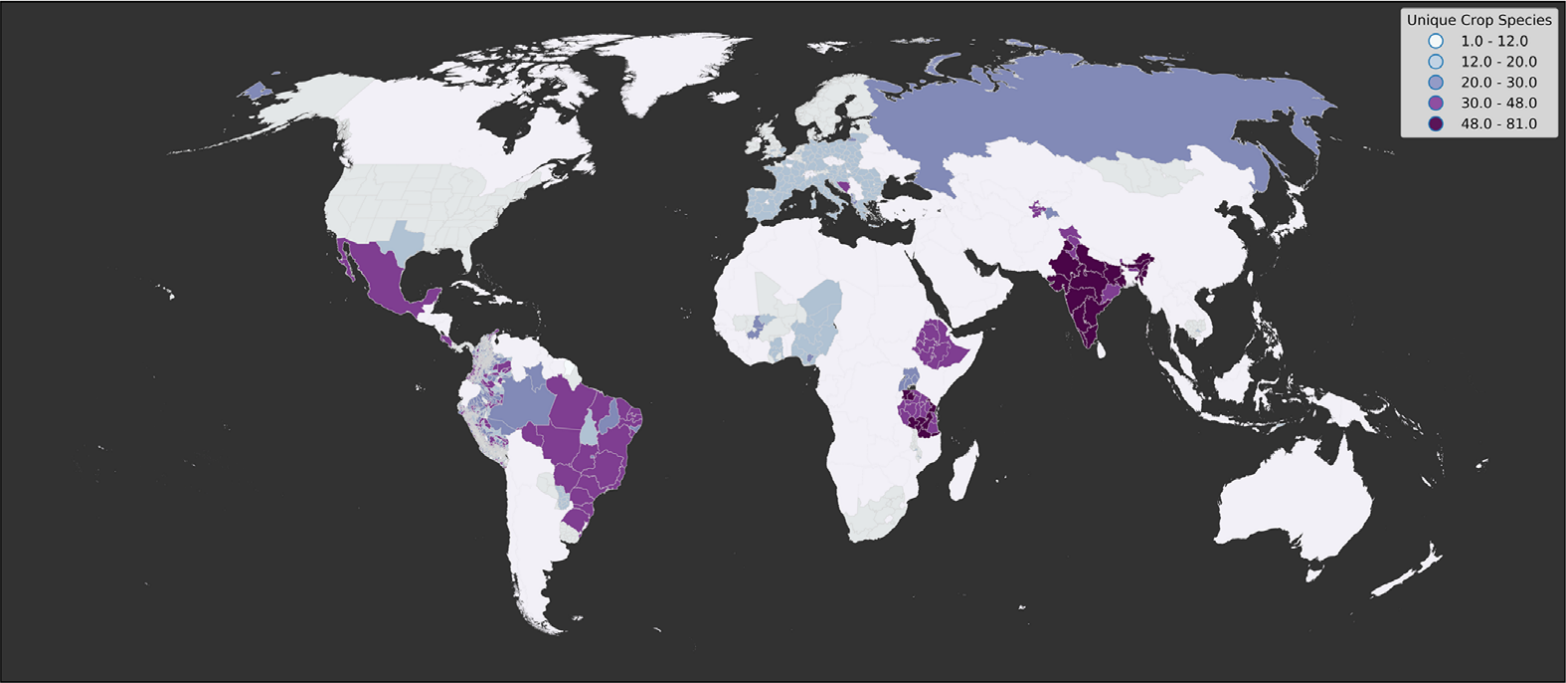
Map showing number of unique crop species per administrate unit at dataset׳s finest resolution.

**Fig. 4:**
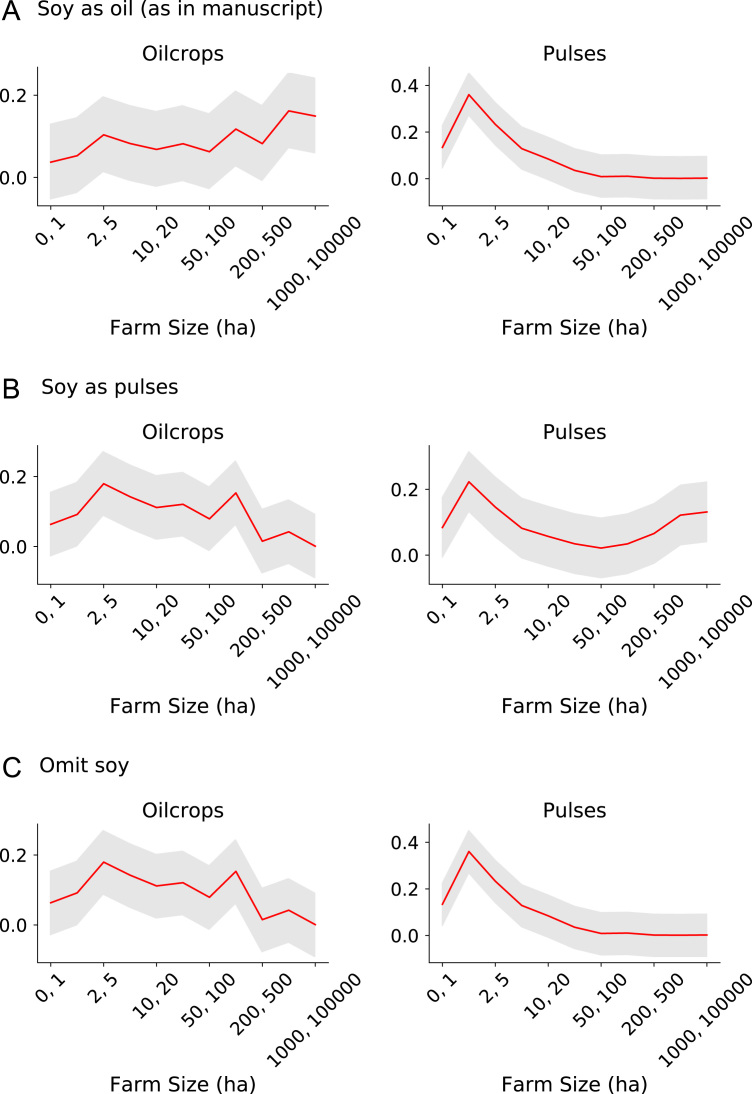
The effect of different classifications of soy on distribution of global production by farm s for oil crops and pulses. Soy was classified as an oil crop (Panel A as in our dataset and FAOSTAT), as a pulse (Panel B), or omitted (Panel C). The *x*-axis shows each farm size class (ha). The *y*-axis shows the percent of global production. The red line is the average percent of production by farm size class. The gray line indicated 95% confidence intervals.

Our dataset includes primary datasets ranging from 2001 to 2015, with a median year of 2013. It includes 55 countries, 45 of which have subnational resolution, 18 of which have fine scale (i.e., farm level) resolution. Fig. 3 shows the data׳s spatial resolution and distribution of the 154 unique crop species represented; on average (mean), there were 30.8 crop species per country (Standard Deviation (SD)=20.3). Crop species were aggregated to major commodity groups according to FAOSTAT definitions of cereals, fruit, oil crops, pulses, roots and tubers, tree nuts, vegetables, and other. Relying on the FAOSTAT classification has its limitations. For example, soy was classified as an oil crop, but it is also a pulse; therefore, this classification should be used as a guideline (Fig. 4). Due to the aggregated nature of a large number of the sources used, we were only able to present gross agricultural area, not net agricultural area or the number of farmers by farm size class.

### 2.2 Key assumptions

#### 2.2.1 Constant yields

**Fig. 5:**
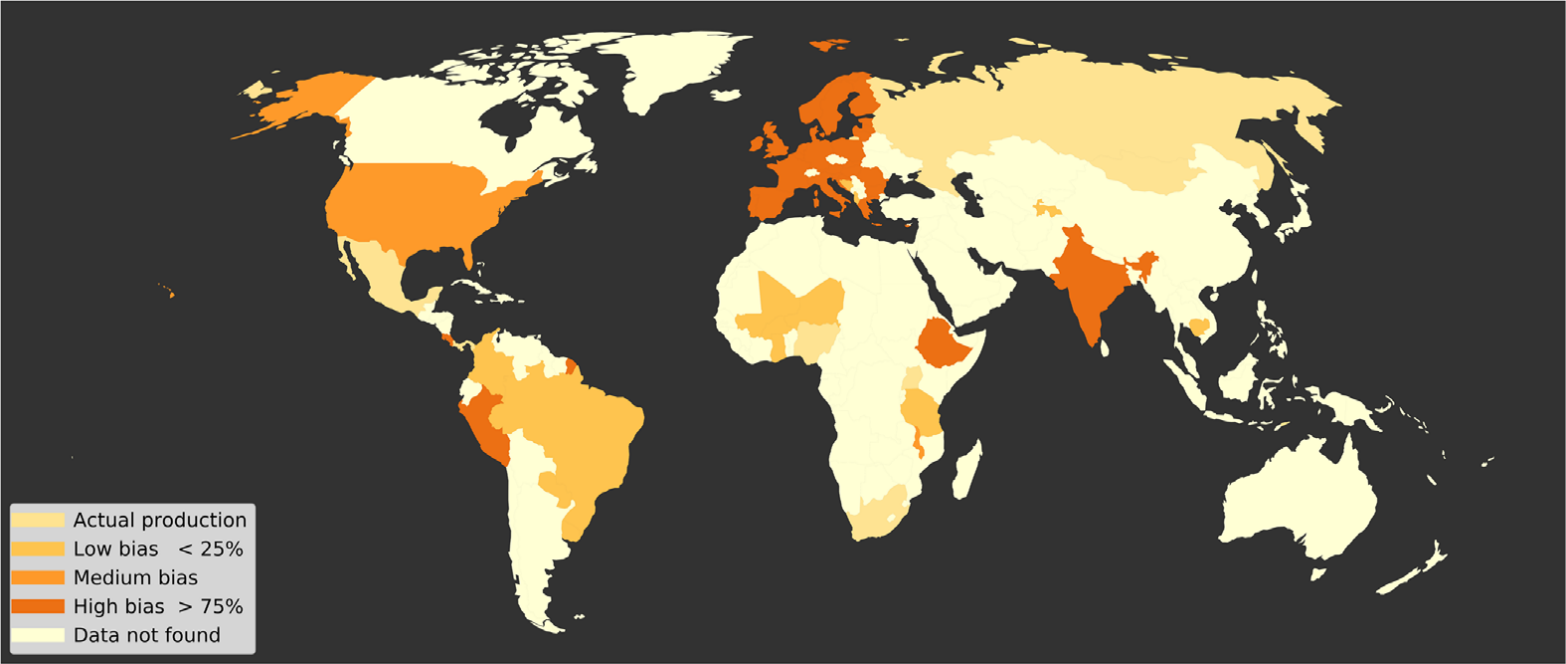
Map showing countries requiring assumption of constant yield across farm sizes. For many countries, our dataset contained a mix of actual production values and only area measurements per crops per farm size; percentages are given for each country according to how much of total crop production was calculated using constant yield assumption (indicated as percent bias in the legend). Darker orange indicates a greater percentage of the country’s data was based on constant yields.

For 33 countries in our dataset, representing 59.7% of the total production (in kcal), we could not find crop production by farm size, but we did find either gross cropped area, harvested area, planted area, or plot area by farm size per crop (Fig. 5). For these data, we used FAOSTAT׳s national yield estimates for the given country, year, and crop to estimate production per farm size. This assumes that all farm sizes within a country had the same yields for a given crop and year. However, as there is a widely observed inverse yield to farm size relationship where smaller farms typically have higher yields [4], [5], [6], we explored how using a constant yield across farm sizes may bias our production estimates.

We tested the presence of a constant yield bias in eight countries for which we had both an area measurement (i.e., harvested, cropped, planted, or plot area) per crop per farm size and crop production by farm size measurement. For these countries, we regressed known production values against production values calculated from constant yields with countries and crop type as random effects, and we report the intercept and slope for this relationship to indicate the level of bias introduced by the constant yield assumption.

**Fig. 6:**
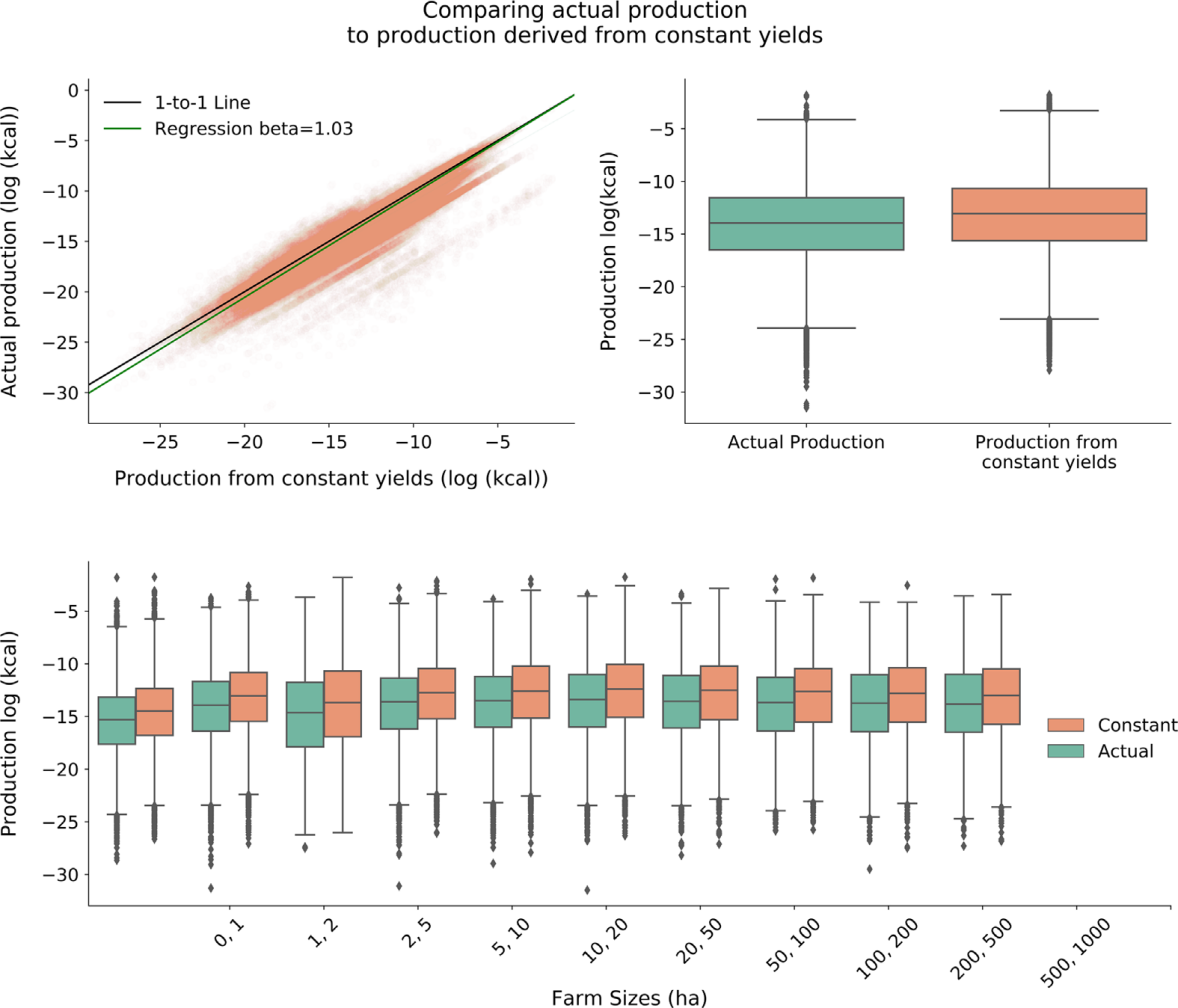
Verifying our constant-yield assumption through comparing production calculated using constant yields versus actual production for countries where we had both area and production data by farm size. A) Log-log plot between constant yield calculated production and actual production. Black line represents 1-to-1 line. Green line is the linear regression line when using constant yield derived production to predict actual production. B) Compares production using constant yields (orange) to actual (green) production on a log-scale, while C) shows this relationship for each farm size class.

Fig. 6A is a log-log plot that shows a high correlation between production computed using constant yields and actual production. We used the natural log of production values to plot this due to long-tailed distributions in the data. We found that using constant yields slightly overestimates actual production for administrative units with smaller production but converges at administrative units with larger production (Intercept: −0.79, SE=0.11; Slope: 1.03, SE=0.001). This bias can be corrected for by predicting out of the model shown in Table 2. In Fig. 6B, we also show boxplots to illustrate this overestimation for all farm size classes, and in Fig. 6C we show the differences for each farm size. The plots indicate that overestimation of production from using constant yield is higher for smaller farm sizes, which is expected due to their higher yields; in general, the FAO yields were higher than the reported yields in our dataset (see section 2.2.2 for details).

**Table 2:** Constant yields at the national level were used to calculate production from cropping area at the sub-national level, then predict actual production. A mixed model was used to account for within country random effects.

	Coef.	Std. Err.	95% CI
Dependent Variable: Actual Production			
Intercept	−0.786*	0.112	−1.005 to −0.567
Production from Constant Yields	1.028*	0.001	1.026 to 1.03
Group RE	4.771	0.484	
N Observations	95850		
N Groups	395		
BIC Full Model	212369.2		
BIC Without Constant Yields	455736.8		

* *p*<0.01.

Where country level yields were not available for certain crops and/or years, regional or global yields were used. Regional and global yields were used for 0.02% of all administrative units in our dataset (and had a Spearman rank correlation of 0.86 with the FAO country level yields) and so we expect them to have small effects on production values estimated across the sample. These are included in the constant yields assumption and the above bias analysis, and the use of constant yields are denoted in the dataset for future researchers.

#### 2.2.2 Calibrating with FAOSTAT

**Fig. 7:**
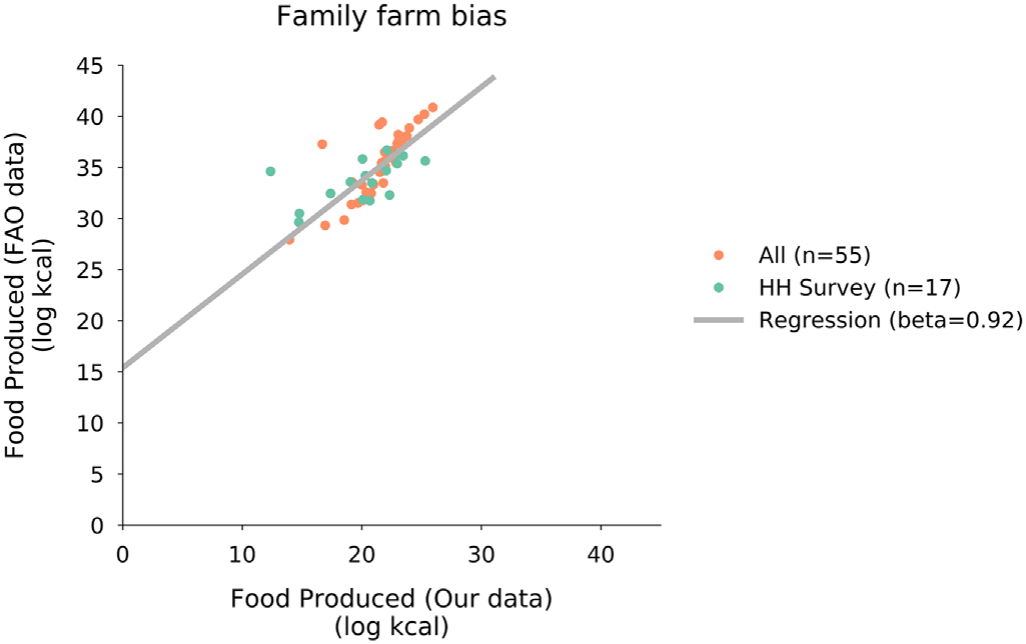
Log–log plot comparing FAOSTAT production values (summed kcal crop equivalents per country) to our dataset with and without household surveys. Household surveys are in green, census data are in orange. The simple linear regression line shows the relationship between the summed production values for countries in our dataset with their FAOSTAT summed production values.

To calibrate our dataset with FAOSTAT we regressed our estimates of country production against theirs for matching crops and years. Our data consistenly underestimates production relative to FAOSTAT (Intercept: 15.39, SE=1.67, and Slope: 0.92, SE=0.08; Fig. 7). This relationship can be used to calibrate our data against FAOSTAT for future researchers interested in using this data. As we used the exact matching of crop lists with the FAO, this is perhaps surprising. It is possible that some of this variation represents differences in survey instruments since we have included different datasets from what FAOSTAT included since we needed to have access to crop production by farm size and FAOSTAT did not provide this cross-tabulation. Another way of looking at this discrepancy is that our dataset provides an independent, and transparent, estimate of the amount of crops produced by different countries across the world.

#### 2.2.3 Family farms bias

For 17 countries in our dataset, representing 22.5% of the total production (in kcal), we could not find agricultural census data, but we did find nationally representative (often with sub-national resolution) agricultural household surveys (Fig. 1). One bias that stems from household surveys is that they only capture family farms, which are often associated with smaller farms. The household surveys miss non-family commercial enterprises and thus do not represent the full population of farms in a country. A proper test of the bias introduced by use of household surveys would require both census and household survey data for the same countries, which we did not have access to for the countries in our dataset and they covered different ranges and magnitudes of production (e.g. with household survey data covering countries with smaller aggregate production; see Fig. 7).

#### 2.2.4 Plot size as a farm size proxy

**Fig. 8:**
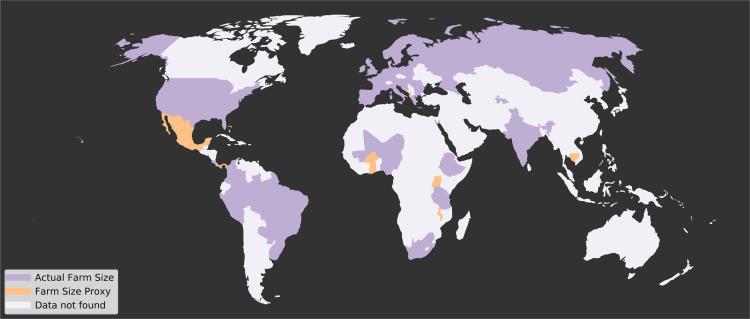
Map showing direct farm size data (purple) or farm size proxy (orange) at the country level.

For 8 countries in our dataset, representing 4.8% of the total production (in kcal), farm size was not explicitly reported, so we calculated a proxy farm size using the sum of either harvested, cropped, planted, or plot area (Fig. 8). This assumption may influence estimates of global crop production by farm size by underestimating farm areas in some farm size classes, because the aggregation process did not capture all fallow plots, water sources, unused areas, and on-farm structures. We think the main effect of this would be to introduce noise into the production by farm size signal (by mixing data using the field size proxy with real farm sizes). Due to data constraints, we were not able to explore how much noise this introduced. It does stand to reason that larger fields need to belong to larger farms, but it is unclear whether smaller fields are part of a large farm with several small fields or part of a small farm. However, because these countries represent less than 5% of the total production covered in our dataset, they do not greatly influence gross estimates of crop production by farm size estimated from these data. When the 8 countries we used a proxy indicator for farm size are omitted from the dataset there was minimal influence on the distribution of food production by farm size (mean absolute difference=0.26; SD=0.19).

#### 2.2.5 Regional bias

**Fig. 9:**
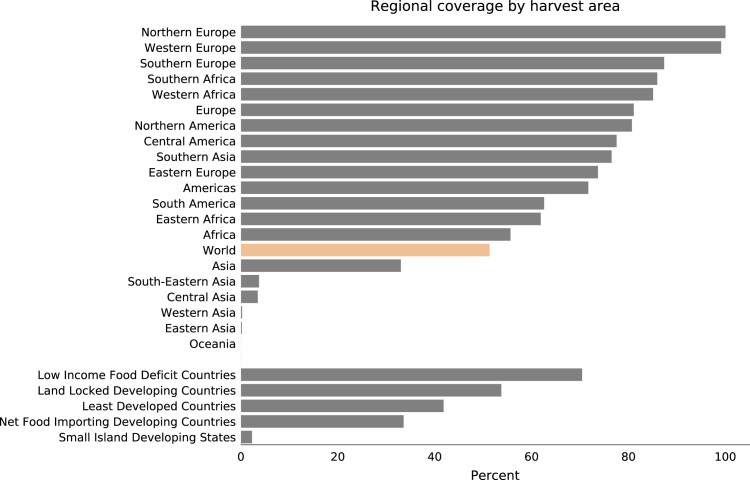
Dataset׳s percent of harvest area by region or economic status compared to global coverage in orange. Harvest area per region calculated from FAOSTAT.

Our dataset accounts for around 51% of the total global harvest area, with representation across country types (e.g., spatial and economic). However, since our dataset is a convenience sample, we were not able to control for spatial coverage nor the countries included, and there were large data gaps for Australasia and Asia (Fig. 9).

**Fig. 10:**
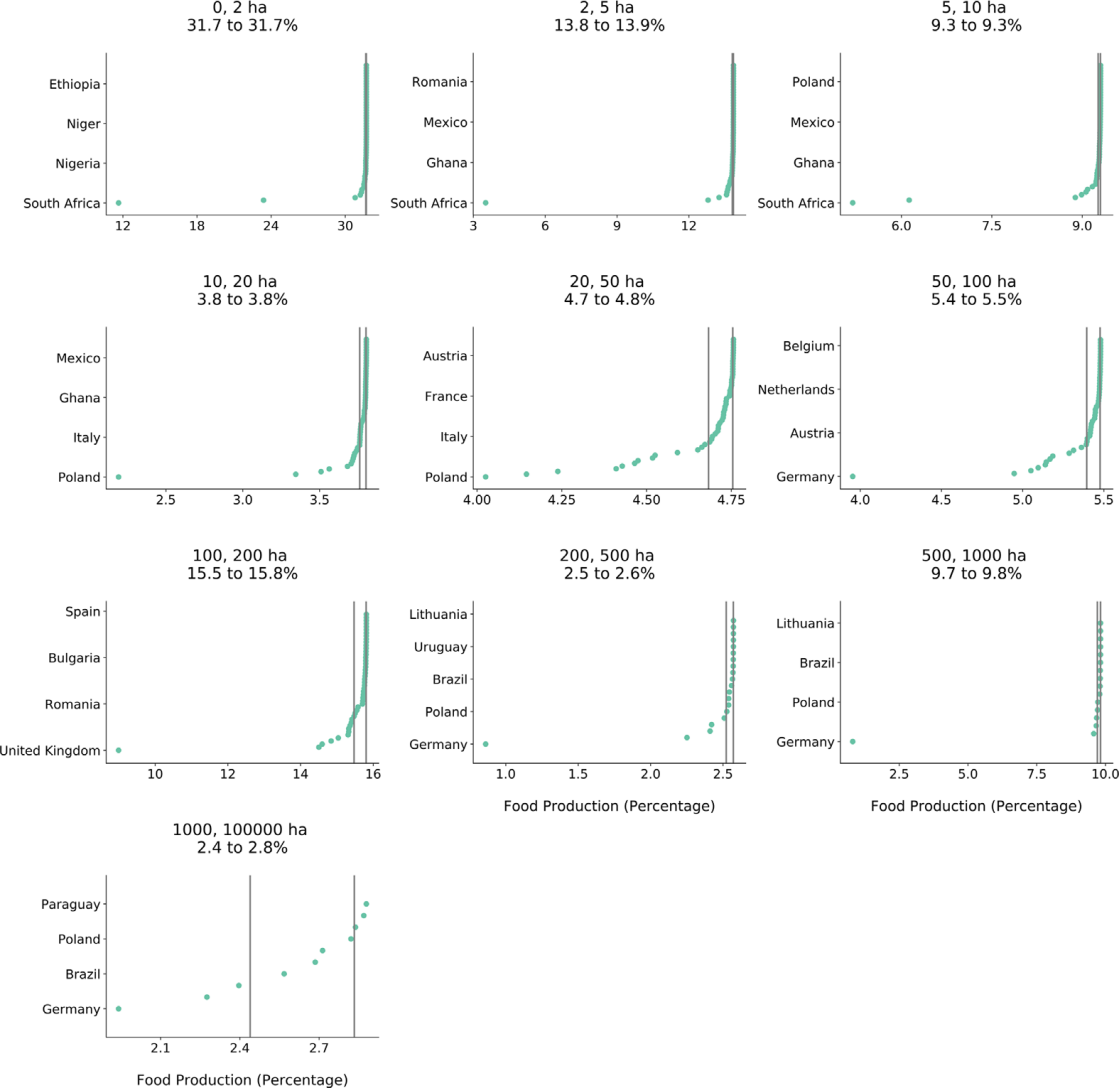
Jackknife plots per farm size to estimate country level bias. Grey lines indicate upper and lower quartiles of global production, and green points refer to the global mean if the country was omitted.

An important question for researchers interested in this dataset is how much the global estimates of crop production by farm size are influenced by the omission of particular countries. While this coverage error is difficult to compute directly, we can explore how sensitive global estimates are to any one country included in the dataset. To do this we re-computed jackknife samples, where one country was omitted with each iteration, shown in Fig. 10. The vertical black line is the mean kilocalories (kcal) of food produced for a given farm size class when no countries were omitted. Each blue dot represents the mean when a corresponding country was omitted. If a country is to the left of the black line it lowers the global average. The vertical lines are the upper and lower quartiles for food production. For each plot, we labelled four countries as examples, but all countries are present.

**Fig. 11:**
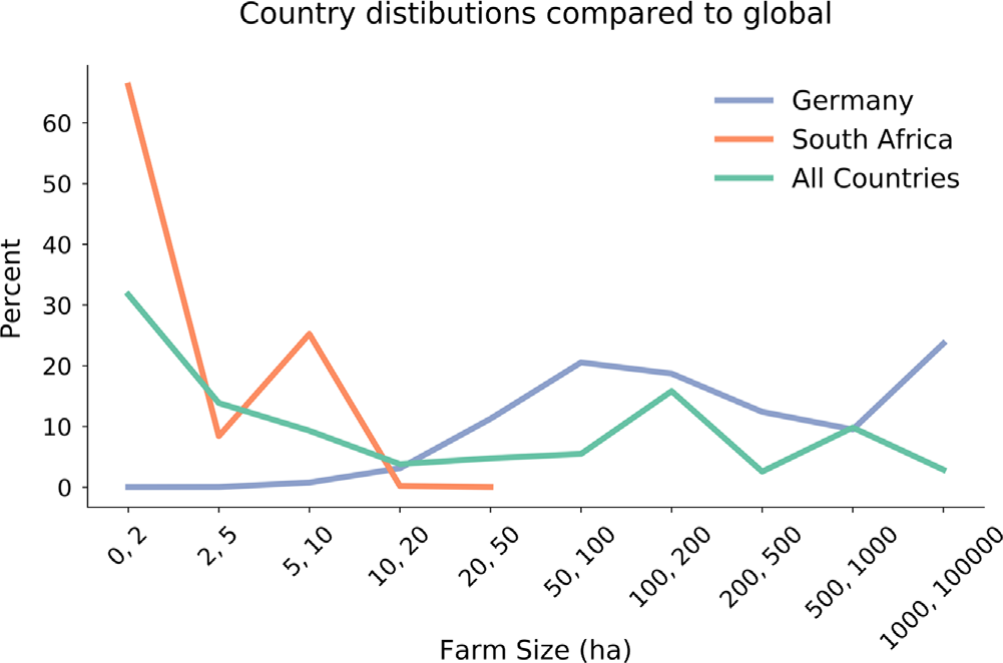
Two examples of countries that deviated from the global distribution of total crop production by farm size: Germany (purple) and South Africa (orange) have different distributions than the global average (green).

There is substantial variation when a country is omitted indicating that countries’ farm size distributions can heavily influence the global averages (see Table 3, Table 4, Table 5 for per country distributions of gross agricultural, total production (kcal), and food production (kcal)). This high variation in the percentage of food produced in different farm size classes indicates that the relationship between farm size and food production is highly contextual; Fig. 11 shows two examples, South Africa and Germany.

**Table 3:** Gross agricultural area (ha 10e5) per country by farm size class (ha).

Country	0 to 1	1 to 2	2 to 5	5 to 10	10 to 20	20 to 50	50 to 100	100 to 200	200 to 500	500 to 1000	1000 to 100000
Albania	6.99	7.19	7.07	5.99	4.82	0.36	0.68	0.00	0.00	0.00	0.00
Austria	0.00	0.06	0.18	0.43	1.33	4.44	3.55	1.97	0.00	0.00	0.00
Belgium	0.00	0.00	0.05	0.16	0.49	1.95	2.23	1.86	0.00	0.00	0.00
Bosnia and Herzegovina	1.07	0.97	2.60	1.25	0.25	0.04	0.01	0.00	0.00	0.00	0.00
Brazil	2702.14	2851.56	2665.63	2580.09	2574.86	2602.58	2649.51	2242.34	1627.48	1128.67	11833.74
Bulgaria	0.51	0.00	0.46	0.44	0.56	1.25	1.38	27.29	0.00	0.00	0.00
Burkina Faso	0.41	2.37	11.13	15.48	16.83	27.06	16.00	15.87	17.64	4.84	43.25
Cambodia	615.54	667.41	698.93	652.37	416.22	281.27	36.16	65.84	31.80	0.00	0.00
Colombia	1.47	7.47	0.00	7.16	7.83	8.71	4.90	3.58	3.66	2.32	3404.38
Costa Rica	0.04	0.10	0.33	0.35	0.36	0.42	0.21	2.32	0.00	0.00	0.00
Croatia	0.21	0.21	1.02	1.03	0.99	1.43	1.13	2.43	0.00	0.00	0.00
Cyprus	0.07	0.07	0.10	0.06	0.06	0.09	0.06	0.11	0.00	0.00	0.00
Denmark	0.00	0.00	0.00	0.17	0.50	1.79	2.78	13.59	0.00	0.00	0.00
Estonia	0.00	0.00	0.01	0.03	0.07	0.18	0.25	3.69	0.00	0.00	0.00
Ethiopia	504.75	508.39	509.47	437.37	245.63	245.63	0.00	0.00	245.63	0.00	0.00
Finland	0.00	0.00	0.02	0.15	0.70	3.30	4.30	4.30	0.00	0.00	0.00
France	0.00	0.21	0.68	1.21	2.99	12.01	31.24	91.16	0.00	0.00	0.00
Germany	0.02	0.05	0.15	1.25	4.21	12.93	21.54	20.00	13.27	10.17	25.30
Ghana	360.21	366.09	390.96	305.08	266.55	131.01	15.58	19.13	0.00	0.00	0.00
Greece	0.00	2.46	4.53	4.64	4.48	4.67	1.50	0.62	0.00	0.00	0.00
Hungary	0.35	0.35	0.95	1.28	2.02	3.48	3.25	21.23	0.00	0.00	0.00
India	52828.97	54462.16	53805.77	48538.17	43063.67	36568.16	36568.16	0.00	0.00	0.00	0.00
Ireland	0.00	0.00	0.00	0.03	0.12	0.70	1.17	1.51	0.00	0.00	0.00
Italy	1.17	1.72	7.08	8.18	10.07	14.61	9.57	11.34	0.00	0.00	0.00
Latvia	0.00	0.03	0.09	0.17	0.34	0.59	0.64	5.85	0.00	0.00	0.00
Lithuania	0.00	0.11	0.56	0.72	0.93	1.56	2.01	2.36	2.36	5.54	0.00
Luxembourg	0.00	0.00	0.00	0.01	0.01	0.04	0.15	0.29	0.00	0.00	0.00
Malawi	7.71	7.46	3.17	0.00	0.00	0.15	0.00	0.00	0.00	0.00	0.00
Mali	181.76	201.31	141.98	122.02	161.69	186.99	231.78	149.35	213.20	92.49	0.00
Malta	0.00	0.00	0.01	0.00	0.00	0.00	0.00	0.00	0.00	0.00	0.00
Mexico	137.41	136.77	144.82	135.23	127.21	125.76	97.99	73.33	0.00	106.35	0.00
Mongolia	0.00	0.00	0.00	0.00	1.15	1.42	1.31	1.45	1.55	1.49	0.00
Netherlands	0.00	0.01	0.07	0.22	0.57	1.93	2.24	1.90	0.00	0.00	0.00
Niger	47.04	46.84	49.73	0.00	0.00	40.37	0.00	0.00	0.00	0.00	0.00
Nigeria	77.76	96.31	137.10	45.39	14.30	0.00	0.00	0.00	0.00	0.00	0.00
Norway	0.00	0.00	0.02	0.12	0.41	1.10	0.85	0.50	0.06	0.00	0.00
Panama	7.29	7.25	12.33	13.34	12.95	14.45	12.27	12.18	12.59	4.68	0.00
Paraguay	264.73	522.04	522.07	525.73	524.53	520.60	489.25	512.46	515.46	487.86	2277.99
Peru	20172.35	22929.58	0.00	19835.52	16653.75	12758.13	7452.44	4687.71	3152.08	1730.04	2400.11
Poland	0.90	1.49	8.96	15.04	20.03	20.60	9.87	3.02	4.99	4.79	9.90
Portugal	0.25	0.57	1.35	1.25	1.32	1.54	1.00	3.85	0.00	0.00	0.00
Romania	3.33	5.51	10.37	5.19	2.73	2.88	2.72	37.59	0.00	0.00	0.00
Russian Federation	36.63	40.55	64.20	64.20	64.20	64.20	64.20	38.80	35.60	0.00	64.15
Slovakia	0.02	0.02	0.11	0.10	0.13	0.25	0.30	10.69	0.00	0.00	0.00
Slovenia	0.05	0.05	0.24	0.32	0.33	0.34	0.13	0.23	0.00	0.00	0.00
South Africa	174.67	238.20	119.08	119.08	85.55	29.39	0.00	0.00	0.00	0.00	0.00
Spain	0.98	0.98	5.29	7.02	10.22	20.23	20.43	52.61	0.00	0.00	0.00
Sweden	0.00	0.00	0.01	0.06	0.25	1.14	2.15	8.65	0.00	0.00	0.00
Tajikistan	0.00	4.73	6.04	6.15	6.16	6.31	5.45	5.95	3.30	1.53	0.00
Timor-Leste	8.81	7.22	6.23	3.86	3.25	0.00	0.96	0.00	0.00	0.00	0.00
Uganda	214.22	194.27	162.79	67.14	27.19	25.77	0.00	0.00	0.00	0.00	0.00
United Kingdom	0.00	0.00	0.01	0.06	0.24	1.74	5.18	36.35	0.00	0.00	0.00
United Republic of Tanzania	1718.75	1773.16	1740.60	1385.95	1091.61	642.99	310.78	253.57	160.63	81.96	739.73
United States of America	0.00	0.00	51.82	49.39	50.56	21.01	51.47	102.88	422.47	661.39	0.00
Uruguay	0.00	61.10	77.83	72.62	132.63	197.44	243.10	245.07	287.27	301.85	884.70

**Table 4:** Total crop production (kcal 10e7) per country by farm size class (ha).

Country	0 to 1	1 to 2	2 to 5	5 to 10	10 to 20	20 to 50	50 to 100	100 to 200	200 to 500	500 to 1000	1000 to 100000
Albania	6.98	13.12	23.41	5.59	3.22	0.03	0.14	0.00	0.00	0.00	0.00
Austria	0.00	0.21	1.77	4.02	23.15	165.87	210.81	113.53	0.00	0.00	0.00
Belgium	0.00	0.14	2.23	7.28	28.81	164.38	240.25	219.82	0.00	0.00	0.00
Bosnia and Herzegovina	20.63	13.55	40.62	17.02	2.17	0.01	0.12	0.08	0.05	0.00	0.00
Brazil	33.41	41.06	92.91	99.78	161.02	243.32	154.50	151.01	207.51	188.54	1188.03
Bulgaria	5.89	0.00	8.54	10.05	13.76	32.19	35.81	713.80	0.00	0.00	0.00
Burkina Faso	3.70	9.44	39.77	52.11	47.30	77.63	52.53	29.88	27.27	8.05	145.15
Cambodia	0.11	0.19	0.39	0.48	0.33	0.22	0.02	0.01	0.02	0.00	0.00
Colombia	6.23	20.67	0.00	22.86	27.44	34.31	21.25	16.01	16.41	11.10	28.87
Costa Rica	0.11	0.31	1.45	2.20	2.34	2.91	2.43	43.31	0.00	0.00	0.00
Croatia	1.11	1.11	8.02	10.37	11.47	20.58	20.24	55.29	0.00	0.00	0.00
Cyprus	0.11	0.11	0.27	0.29	0.38	0.51	0.30	0.29	0.00	0.00	0.00
Denmark	0.00	0.00	0.06	1.66	5.09	25.24	45.75	265.20	0.00	0.00	0.00
Estonia	0.01	0.04	0.20	0.40	0.75	1.69	2.02	29.73	0.00	0.00	0.00
Ethiopia	117.14	80.16	49.31	4.44	0.46	0.23	0.00	0.00	1.61	0.00	0.00
Finland	0.00	0.00	0.11	0.89	4.74	30.30	43.02	49.02	0.00	0.00	0.00
France	0.00	1.65	8.37	16.92	33.63	243.82	990.74	4129.15	0.00	0.00	0.00
Germany	0.04	0.08	0.55	17.43	67.32	233.95	418.15	394.77	261.83	200.78	499.23
Ghana	163.08	181.14	507.81	335.58	202.59	33.14	42.53	55.86	0.00	0.00	0.00
Greece	0.00	14.82	36.84	45.61	52.10	62.24	21.62	9.94	0.00	0.00	0.00
Hungary	2.55	2.55	9.39	13.74	24.47	43.73	42.72	335.13	0.00	0.00	0.00
India	5817.48	4152.57	5168.19	2053.26	802.54	362.14	40.24	0.00	0.00	0.00	0.00
Ireland	0.00	0.00	0.05	0.33	1.73	9.89	19.49	38.55	0.00	0.00	0.00
Italy	9.84	14.51	85.27	116.82	159.41	275.75	176.57	217.21	0.00	0.00	0.00
Latvia	0.00	0.64	1.60	2.66	4.74	7.32	8.41	80.27	0.00	0.00	0.00
Lithuania	0.00	1.92	8.90	11.20	14.38	24.49	34.29	45.46	45.46	106.74	0.00
Luxembourg	0.00	0.00	0.01	0.02	0.07	0.64	3.47	6.94	0.00	0.00	0.00
Malawi	28.87	13.66	7.39	0.00	0.00	1.29	0.00	0.00	0.00	0.00	0.00
Mali	0.00	0.00	0.00	0.00	0.00	0.00	0.00	0.00	0.00	0.00	0.00
Malta	0.03	0.02	0.05	0.02	0.01	0.00	0.00	0.00	0.00	0.00	0.00
Mexico	26.14	87.68	300.90	362.64	117.87	31.76	0.86	0.22	0.00	0.37	0.00
Mongolia	0.00	0.00	0.00	0.00	0.00	0.02	0.01	0.03	0.10	0.08	0.00
Netherlands	0.00	0.07	2.68	9.52	34.31	154.01	215.76	226.60	0.00	0.00	0.00
Niger	7.37	328.12	20.89	0.00	0.00	14.29	0.00	0.00	0.00	0.00	0.00
Nigeria	429.60	342.41	186.76	19.10	3.02	0.00	0.00	0.00	0.00	0.00	0.00
Norway	0.00	0.01	0.10	0.47	1.86	6.99	7.04	4.87	0.58	0.00	0.00
Panama	0.00	0.00	0.02	0.09	0.22	0.81	0.84	0.68	0.58	0.29	0.00
Paraguay	0.77	5.99	17.96	29.66	34.39	20.21	11.04	13.35	26.27	22.52	149.93
Peru	17.48	79.15	0.00	38.17	27.63	22.79	9.16	4.72	5.13	7.94	53.74
Poland	14.94	24.80	125.13	199.56	325.06	393.05	193.89	59.86	99.01	94.96	196.23
Portugal	1.55	3.50	6.59	4.77	5.16	7.24	4.36	15.91	0.00	0.00	0.00
Romania	71.32	117.86	226.95	108.44	58.14	62.79	59.10	813.03	0.00	0.00	0.00
Russian Federation	0.00	0.00	1.61	12.00	25.38	13.15	1.87	0.00	0.00	0.00	10.89
Slovakia	0.38	0.38	1.91	1.64	2.00	3.46	4.47	202.70	0.00	0.00	0.00
Slovenia	0.41	0.41	2.75	3.70	3.78	4.06	1.68	3.47	0.00	0.00	0.00
South Africa	1119.33	5312.17	818.11	2454.33	18.36	1.19	0.00	0.00	0.00	0.00	0.00
Spain	4.79	4.79	26.94	36.29	57.75	147.35	179.60	474.16	0.00	0.00	0.00
Sweden	0.00	0.00	0.15	0.72	3.18	21.86	47.23	238.77	0.00	0.00	0.00
Tajikistan	0.00	0.18	0.71	1.78	5.64	5.32	1.83	1.55	1.22	0.41	0.00
Timor-Leste	0.25	0.01	0.01	0.00	0.00	0.00	0.00	0.00	0.00	0.00	0.00
Uganda	45.70	44.30	17.09	2.07	2.34	1.09	0.00	0.00	0.00	0.00	0.00
United Kingdom	0.00	0.00	0.01	1.31	5.44	42.54	109.78	865.93	0.00	0.00	0.00
United Republic of Tanzania	47.30	82.92	141.09	52.19	27.62	16.38	4.89	6.83	8.33	0.58	5.73
United States of America	0.00	0.00	2.16	6.97	14.17	104.78	264.63	596.45	1703.26	6452.80	0.00
Uruguay	0.00	0.00	0.01	0.07	0.17	1.42	5.33	11.55	46.78	70.40	706.18

**Table 5:** Food production (kcal 10e7) per country by farm size class (ha).

Country	0 to 1	1 to 2	2 to 5	5 to 10	10 to 20	20 to 50	50 to 100	100 to 200	200 to 500	500 to 1000	1000 to 100000
Albania	4.21	7.86	14.22	3.37	1.97	0.02	0.04	0.00	0.00	0.00	0.00
Austria	0.00	0.09	1.17	2.69	18.62	146.49	191.30	102.35	0.00	0.00	0.00
Belgium	0.00	0.08	1.34	4.13	17.00	103.24	151.84	137.79	0.00	0.00	0.00
Bosnia and Herzegovina	12.08	6.32	18.35	7.59	0.94	0.01	0.10	0.04	0.03	0.00	0.00
Brazil	13.08	15.78	28.42	26.07	44.03	73.75	52.36	47.43	67.26	62.85	219.28
Bulgaria	3.38	0.00	5.18	6.18	8.40	19.77	22.01	433.20	0.00	0.00	0.00
Burkina Faso	1.98	5.06	21.29	27.90	25.32	41.56	28.12	15.99	14.60	4.31	77.70
Cambodia	0.07	0.12	0.25	0.31	0.22	0.14	0.01	0.01	0.01	0.00	0.00
Colombia	3.98	14.08	0.00	15.78	19.15	23.90	15.31	12.04	12.59	7.46	22.16
Costa Rica	0.09	0.25	1.16	1.83	1.95	2.45	2.08	36.80	0.00	0.00	0.00
Croatia	0.57	0.57	3.98	5.50	6.76	13.03	14.04	41.52	0.00	0.00	0.00
Cyprus	0.07	0.07	0.17	0.19	0.27	0.36	0.22	0.22	0.00	0.00	0.00
Denmark	0.00	0.00	0.02	0.80	2.39	12.80	25.13	151.42	0.00	0.00	0.00
Estonia	0.01	0.03	0.12	0.21	0.36	0.78	0.86	12.38	0.00	0.00	0.00
Ethiopia	106.71	72.72	44.65	4.03	0.43	0.22	0.00	0.00	1.52	0.00	0.00
Finland	0.00	0.00	0.06	0.51	2.91	20.68	29.60	34.68	0.00	0.00	0.00
France	0.00	0.82	4.35	10.35	19.51	154.53	681.26	3045.30	0.00	0.00	0.00
Germany	0.02	0.05	0.30	8.90	36.05	130.13	237.32	216.04	143.29	109.88	273.21
Ghana	74.93	85.91	267.46	186.95	107.91	19.97	26.13	22.28	0.00	0.00	0.00
Greece	0.00	9.34	25.18	32.32	38.24	46.90	16.25	7.40	0.00	0.00	0.00
Hungary	1.54	1.54	5.86	8.58	15.49	27.64	27.35	224.59	0.00	0.00	0.00
India	5239.30	3718.33	4620.46	1836.08	720.24	326.47	36.27	0.00	0.00	0.00	0.00
Ireland	0.00	0.00	0.02	0.17	1.01	5.37	10.92	20.79	0.00	0.00	0.00
Italy	7.57	11.17	69.00	95.70	131.67	231.40	148.96	184.61	0.00	0.00	0.00
Latvia	0.00	0.23	0.56	0.91	1.57	2.36	2.65	27.24	0.00	0.00	0.00
Lithuania	0.00	0.83	3.71	4.59	5.80	9.72	13.99	20.30	20.30	47.67	0.00
Luxembourg	0.00	0.00	0.01	0.01	0.04	0.38	2.30	4.44	0.00	0.00	0.00
Malawi	14.93	7.58	4.61	0.00	0.00	0.86	0.00	0.00	0.00	0.00	0.00
Mali	0.00	0.00	0.00	0.00	0.00	0.00	0.00	0.00	0.00	0.00	0.00
Malta	0.02	0.01	0.03	0.01	0.00	0.00	0.00	0.00	0.00	0.00	0.00
Mexico	12.14	40.55	129.45	140.39	54.80	15.00	0.40	0.10	0.00	0.17	0.00
Mongolia	0.00	0.00	0.00	0.00	0.00	0.01	0.01	0.02	0.07	0.06	0.00
Netherlands	0.00	0.05	2.10	7.60	28.09	125.75	171.36	177.20	0.00	0.00	0.00
Niger	5.12	213.91	14.41	0.00	0.00	9.80	0.00	0.00	0.00	0.00	0.00
Nigeria	230.53	175.49	104.30	11.85	1.83	0.00	0.00	0.00	0.00	0.00	0.00
Norway	0.00	0.00	0.05	0.28	1.11	4.43	4.57	3.19	0.38	0.00	0.00
Panama	0.00	0.00	0.02	0.08	0.21	0.78	0.80	0.65	0.56	0.28	0.00
Paraguay	0.32	2.92	8.77	15.22	18.16	11.60	6.36	7.42	14.60	12.43	90.16
Peru	8.53	42.97	0.00	21.83	16.17	13.53	5.54	2.94	2.82	4.27	29.71
Poland	7.31	12.13	61.58	107.36	205.10	272.68	138.73	43.53	72.01	69.06	142.71
Portugal	0.72	1.63	3.40	2.95	3.44	4.56	2.64	8.71	0.00	0.00	0.00
Romania	32.47	53.66	119.31	59.75	33.50	37.59	37.15	543.19	0.00	0.00	0.00
Russian Federation	0.00	0.00	1.26	8.34	17.42	9.20	1.32	0.00	0.00	0.00	8.09
Slovakia	0.16	0.16	0.79	0.67	0.80	1.37	1.98	116.37	0.00	0.00	0.00
Slovenia	0.21	0.21	1.33	1.87	2.02	2.19	0.89	1.75	0.00	0.00	0.00
South Africa	646.65	3070.00	472.58	1417.75	11.93	0.69	0.00	0.00	0.00	0.00	0.00
Spain	3.43	3.43	18.88	25.23	40.72	106.52	131.32	345.02	0.00	0.00	0.00
Sweden	0.00	0.00	0.13	0.52	2.37	17.86	39.05	200.04	0.00	0.00	0.00
Tajikistan	0.00	0.11	0.43	1.06	3.36	3.18	1.10	0.93	0.74	0.25	0.00
Timor-Leste	0.23	0.01	0.01	0.00	0.00	0.00	0.00	0.00	0.00	0.00	0.00
Uganda	32.52	30.83	12.71	1.57	1.74	0.73	0.00	0.00	0.00	0.00	0.00
United Kingdom	0.00	0.00	0.01	0.88	3.66	28.71	74.18	583.27	0.00	0.00	0.00
United Republic of Tanzania	32.23	56.26	95.09	35.17	18.62	11.04	3.77	4.51	5.63	0.40	3.72
United States of America	0.00	0.00	0.89	2.75	5.60	31.07	86.21	216.30	764.14	4023.73	0.00
Uruguay	0.00	0.00	0.01	0.04	0.10	0.85	3.20	6.95	28.11	42.26	422.63

## Footnotes

1 Where there was no crop production by farm size data available we extracted farm size by either harvest area, cultivated area, crop area, or planted area. We will refer to this as ‘cropping area’ in this article.
